# Sediment supply dampens the erosive effects of sea-level rise on reef islands

**DOI:** 10.1038/s41598-021-85076-x

**Published:** 2021-03-09

**Authors:** Megan E. Tuck, Murray R. Ford, Paul S. Kench, Gerd Masselink

**Affiliations:** 1 School of Environment, University of Auckland, Private Bag 92019, Auckland, New Zealand; 2 Department of Earth Sciences, Simon Fraser University, Burnaby, BC Canada; 3 School of Biological and Marine Sciences, University of Plymouth, Plymouth, PL4 8AA UK

**Keywords:** Climate sciences, Environmental sciences, Natural hazards

## Abstract

Large uncertainty surrounds the future physical stability of low-lying coral reef islands due to a limited understanding of the geomorphic response of islands to changing environmental conditions. Physical and numerical modelling efforts have improved understanding of the modes and styles of island change in response to increasing wave and water level conditions. However, the impact of sediment supply on island morphodynamics has not been addressed and remains poorly understood. Here we present evidence from the first physical modelling experiments to explore the effect of storm-derived sediment supply on the geomorphic response of islands to changes in sea level and energetic wave conditions. Results demonstrate that a sediment supply has a substantial influence on island adjustments in response to sea-level rise, promoting the increase of the elevation of the island while dampening island migration and subaerial volume reduction. The implications of sediment supply are significant as it improves the potential of islands to offset the impacts of future flood events, increasing the future physical persistence of reef islands. Results emphasize the urgent need to incorporate the physical response of islands to both physical and ecological processes in future flood risk models.

## Introduction

The future physical stability of coral reef islands and the continued existence of atoll nations is widely considered at risk from the impacts of anthropogenic climate change, particularly eustatic sea-level rise (SLR)^1,2^. Low-lying accumulations of unconsolidated calcareous sediment, organized on reef platforms by hydrodynamic processes, reef islands are considered extremely vulnerable to physical destabilization as a result of projected increases in sea level^3,4^ and changing wave conditions^5,6^. In addition to shoreline erosion through increased wave attack, the future stability of reef islands is also expected to be largely impacted by alterations to island sediment supply^7^. Composed entirely of carbonate sediment, reef islands are reliant on the delivery of sediment from the surrounding reef for island formation and future development^7,8^. Therefore, the future stability of islands is expected to be highly sensitive to changes in ecological processes that may modify coral reef community composition and subsequent sediment generation^7,9^. However, despite the critical control sediment transport processes have on reef island maintenance and morphological stability, there is limited understanding of how these processes may change under future rising sea levels and the consequential effect on island morphology.

Reef islands, typically located in mid-ocean settings within tropical and sub-tropical oceans, provide the only habitable land in atoll nations of Kiribati, Maldives, Marshall Islands, Tokelau and Tuvalu. Several classification schemes have been developed to explain the difference in atoll islands based on their morphology, sedimentary composition and position on the atoll rim^10,11^. Under the simplest classification, reef islands can be divided into two basic types; sand cays and gravel *motu*^10,11^. Sand cays are low-lying, typically ~ 2 m above sea level and generally symmetrical or oval in planform-shape and are often found on the leeward side of atolls. In contrast *motu* are associated with high energy environments and are generally located on the windward side of coral atolls. *Motu* are typically narrow and elongate islands exhibiting a high oceanward gravel ridge, a lower and flatter lagoonward shoreline and the ends of the island are commonly characterised by low-lying spits curved lagoonward^10^. *Motu* develop through the episodic deposition of coarse gravel cobble-sized carbonate sediment onto the reef platform by high energy storm events.

The ongoing security of reef island nations is strongly linked to the future stability and persistence of reef islands. A number of modelling studies exploring the impact of future SLR on reef islands indicate they will be rendered uninhabitable within the next couple of decades as a result of physical destabilization through wave erosion^12^, an increase in the frequency and magnitude of wave-driven flooding^2,13,14^ and saline intrusion of fresh groundwater reserves^15^. However, assertions of extreme and immediate island vulnerability to SLR are often founded on the results of flood risk assessments that simulate future sea levels on present-day island topography^2,13,14,16^. These studies treat reef islands as geomorphologically inert structures, despite evidence that many islands have undergone significant changes in size, shape and position on the reef platform in response to changing sea level, wave conditions and sediment supply, over a range of spatio-temporal scales^8,17–19^.

Recent studies have adopted physical modelling methods and have quantified a range of mechanisms and styles of island change identifying: vertical increases in island elevation, lagoonward island migration and spit rotation as the key physical responses of islands to rising sea levels^20,21^. These studies have furthered the understanding of island morphodynamics, presenting the first experimental evidence that reef islands can offset future flood events through vertical island building and potentially keep pace with SLR^20^. Physical modelling results have been corroborated by numerical modelling studies which explore island response to a wider range of SLR scenarios similarly concluding that island crest buildup in response to SLR can maintain island surfaces above sea level^22^. However, both physical and numerical modelling results reflect conditions in which islands are composed of a fixed volume of sediment. As a result, the islands are constrained to a certain range of responses which conserved the island volume thus precluding the effect of factors, such as sediment supply, that may offset the physical response of islands to changing wave and water level conditions.

A shift in reef island sediment supply through either a change in sediment production on the reef flat, or through changes in the rate and magnitude of sediment transport is expected to have a large control over the development and morphological stability of reef islands^7^. However, limited understanding of the effect of changes in sediment supply on reef island morphodynamics renders large uncertainty surrounding the future morphological trajectory of islands^7^. Understanding the role sediment supply plays in governing geomorphic island adjustments is crucial, particularly the height of the island above sea level. This height, known as island freeboard, controls the frequency and magnitude of overwash processes and consequently, future flood risk to island communities^23^.

Here we present results of a series of experiments which use a 1:50 scaled physical model of a gravel *motu* to investigate the effect of changes in sediment supply on the morphological adjustment of atoll islands to increasing sea level (0.5 m and 1 m) under energetic wave conditions (3 m and 4 m). The sediment supply simulated is analogous of an episodic storm-derived sediment supply, a key characteristic of *motu* morphodynamics^24^, rather than a continuous gradual input of sediment (Supplementary Material S1). These experiments enable exploration of the impact of sediment supply on reef island morphodynamics and provide new insights into the role sediment generation may play in the future susceptibility of islands to wave-driven flooding. All dimensional references mentioned in this paper are at prototype scale, unless otherwise stated.

## Results

**Figure 1:**
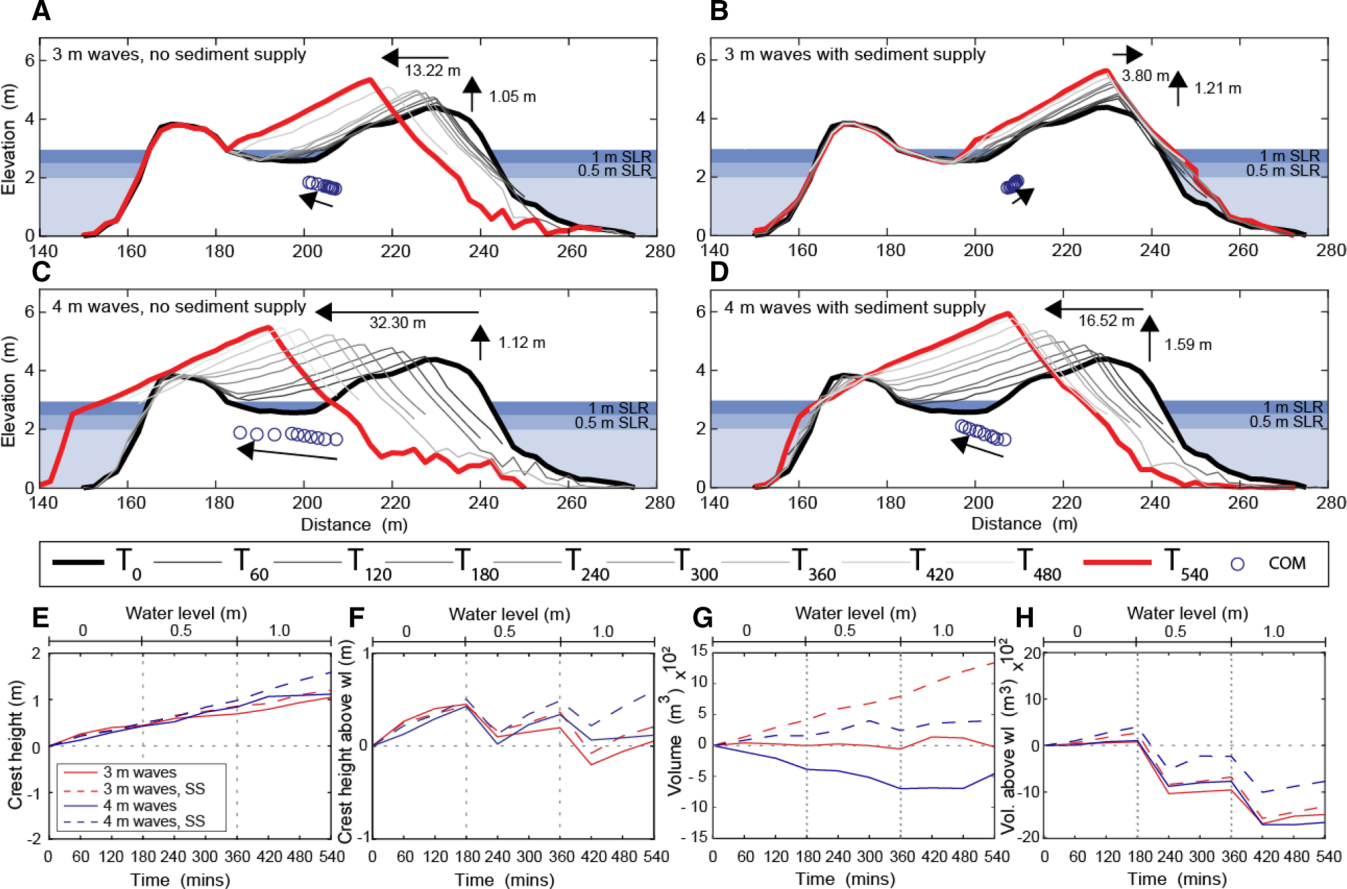
Results of flume experiments with 0 m (0–180 min), 0.5 m (180–360 min) and 1.0 m (360–540 min) SLR under 3 m (**A**,**B**) and 4 m (**C**,**D**) wave conditions without and with sediment supplied to the island, respectively. Circles show displacement of the islands center of mass (the net outcome of both horizontal and vertical island adjustments) across the simulations. Values of vertical accretion and shoreline lateral displacement during each experiment are depicted in A-D. Changes in crest height (**E**), crest height above wl (**F**), Island volume (**G**) and Island volume above wl (H) across the simulations is presented.

**Figure 2:**
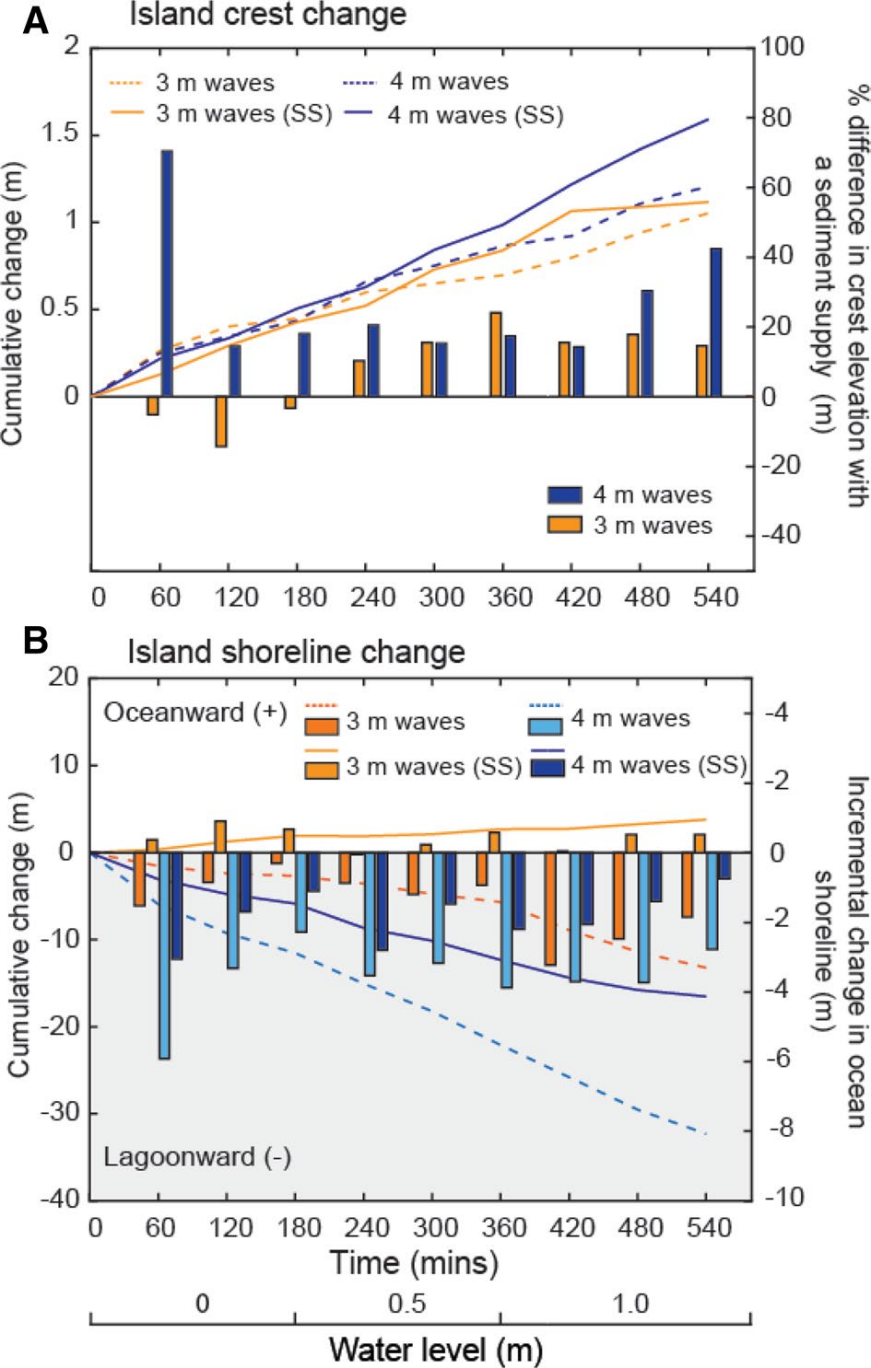
Comparison of changes in (**A**) ocean crest elevation, and (**B**) shoreline migration, under 3 m and 4 m waves with and without a sediment supply. (**A**) Cumulative change in crest elevation across each simulation (left axis) and the relative effect of sediment supply on crest growth at each time-step (right axis) under 3 m and 4 m waves. (**B**) Cumulative shoreline movement across each simulation (left axis) and incremental shoreline change at each time-step (right axis) with and without a sediment supply under 3 m and 4 m waves. There is no change in crest accretion and shoreline erosion at T_0_.

During all physical modelling experiments, the island exhibited both lateral and vertical adjustments in response to SLR. Without the addition of sediment, the oceanward crest increased in elevation by 1.05 m and 1.12 m when exposed to 3 m and 4 m waves and 1 m SLR (T_540_), respectively (Fig. 1A,C). Significantly, these increases in crest elevation enabled the island to maintain and increase freeboard under both 3 and 4 m wave conditions (Fig. 1E,F). Maximum increases in crest elevation occurred when sediment was added to the island; crest height increased by 1.21 m and 1.59 m after 1 m SLR (T_540_) increasing island freeboard by 0.16 m and 0.47 m, when exposed to 3 m and 4 m waves, respectively (Fig. 1B,D,F). The change in the elevation of the island crest was on average 8% and 27% greater when sediment supply was added than at the comparable time during experiments without sediment supply when exposed to 3 and 4 m waves, respectively (Fig. 2).

**Figure 3:**
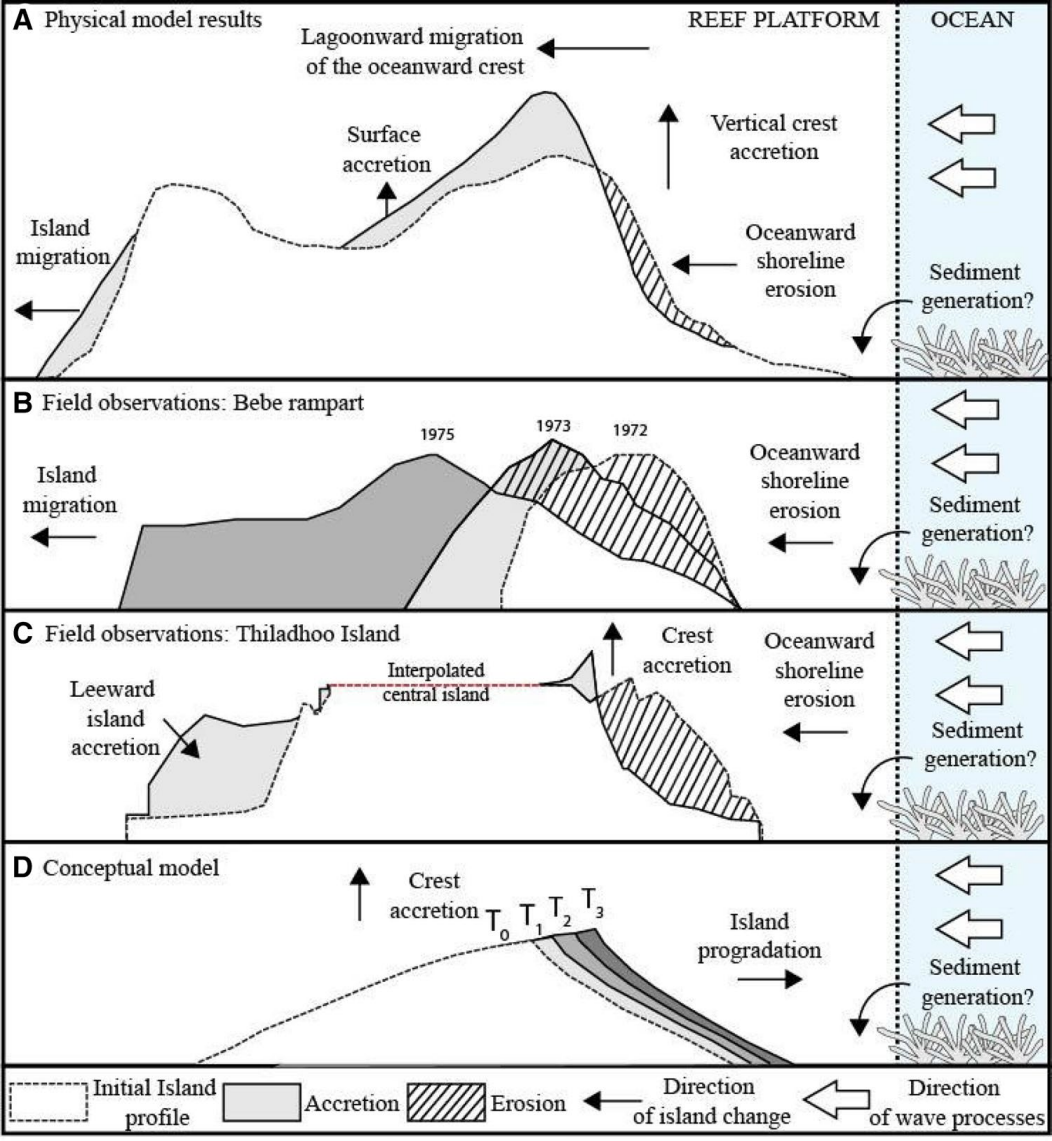
A conceptual model of island response to changing boundary conditions with a sediment supply observed in (**A**) the wave flume, (**B**,**C**) Field observations^30,32^, and (**D**) conceptual model^7^.

In addition to accelerating the rate of increase in crest elevation, the addition of sediment to the island shoreline reduced lagoonward migration of the island under all wave and water level conditions (Figs. 2, 3). During experiments without sediment supply, the ocean shoreline migrated 13.2 m and 32.3 m under 3 m and 4 m waves and 1 m SLR (T_540_) (Fig. 1A,C). However, when exposed to 3 m waves no lagoonward migration was observed when sediment was added to the island; instead the ocean shoreline prograded 3.8 m (Fig. 2B). When exposed to 4 m waves lagoonward island migration was reduced to 16.5 m when a sediment supply was simulated (Fig. 1D). Lagoonward island migration under 4 m waves was on average by 54% less when sediment was added to the island than at the comparable time during the experiment without sediment supply (Fig. 2). As a result of both vertical and planform island adjustments with SLR, the subaerial volume of the island reduced (Fig. 1G,H). However, reductions were 12% and 53% less when additional sediment was added to the flume under 3 m and 4 m waves, respectively. Although added sediment dampened the erosive effects of rising water levels, the volume of the island above water level was still reduced by 39% and 23% when exposed to 1 m of SLR under 3 m and 4 m waves (Fig. 1H).

## Discussion and conclusions

A robust understanding of coral reef island morphodynamics in response to shifts in sediment supply is critical for accurately predicting future island trajectories^7^. Our results demonstrate the substantial role sediment supply plays in the control of future geomorphic island adjustments in response to SLR. Additional sediment added to the island accelerates the rate of increasing crest elevation under both 3 m and 4 m waves, further improving the ability of islands to keep up with SLR. Simultaneously, a sediment supply dampens the erosive effects of SLR, by reducing lagoonward island migration a larger amount of island volume is maintained with rising water levels.

Island building as a result of storm-driven inputs into reef island sedimentary systems has also been observed in the field^17,19,24–26^. Several studies^17,27,28^ have identified storms as a key mechanisms responsible for reshaping reef islands. For example, a typhoon at Jaluit Atoll in the Marshall Islands was responsible for a 0.50 km^2^ instantaneous reduction in island size, while generating massive quantities of sediment which eventually contributed to the growth of the islands to a degree that they exceeded pre-storm area within decades^17^. Similarly, a large swell event deposited up to 5 cm on to the surface of Nukutao Island in Papua New Guinea, contributing the vertical accretion of the island^27^. While having differing hydrodynamic characteristics to storms, the 2004 Sumatran tsunami was responsible for the deposition of up to 30 cm of sediment on the surface of some islands on South Maalhosmadulu Atoll in the Maldives^29,30^. Our results are also consistent with interpretations of geometric and conceptual models that identified sediment budgets as critical to determining island response to rising sea levels (Fig. 3)^7,31^.

The results of previous physical and numerical modelling experiments indicate that under rising sea levels overtopping processes facilitate vertical island building^20–22^. The experiments presented in this paper reveal that when additional sediment is added to the island the rate of vertical crest accretion is enhanced (Figs. 2, 3). A possible mechanism that may explain this is the reduction of lagoonward shoreline displacement when sediment supply is simulated. Lagoonward displacement of the shoreline increases wave energy dissipation, reducing the wave energy available for run-up and therefore sediment transport to the island crest. Modelling results indicate that a sediment supply mitigates lagoonward island migration enabling a higher frequency of washover processes to transport sediment onto the island surface, compared to experiments without sediment added to the island (Fig. 1). The slope of the beach is reduced from 14.3° to ~ 9.7° whether sediment is added to the island or not (Fig. 1). Therefore, change in beach slope is not considered a possible mechanism affecting the ability of islands to increase in crest height when sediment is added to the island.

Sea level and offshore wave conditions both determine whether overwash or overtopping occurs and, therefore, whether sediment deposited on the reef platform during high-energy events is transported to the island crest (overtopping) or onto the island surface (overwash). When exposed to the 3 m waves and future sea levels, overtopping processes deposit additional sediment at the island crest and oceanward beach driving crest accretion and oceanward beach progradation (Fig. 1B). In contrast, under larger (4 m) wave conditions and higher water levels, overwash processes dominate, transporting sediment lagoonward beyond the oceanward crest triggering accretion of the island surface (Fig. 1D).

### Modelling limitations

The physical modelling experiments were designed to explore the effect of sediment supply on the modes and styles of island response to changes in sea level and wave conditions, rather than generating site-specific morphologic predictions. Despite replicating a reef environment that captures more of the processes driving island change than previous modelling studies, there remain a number of limitations to be considered. First, vertical reef growth may further attenuate wave energy reaching the shoreline by altering the roughness of the reef surface^33^, although it is important to note that the future rate of reef growth is uncertain and may not keep up with SLR^23,34^. Second, sea-level rise would be gradual, punctuated by periods of sea-level fall and stability rather than the instantaneous 0.5 m steps simulated in the model^35^. The future SLR adopted in the model are close to 0.98 m, the upper limit of SLR of the IPCC Fifth Assessment Report high emission scenario (RCP 8.5) for the year 2100^35,36^. However, we caution that some projections of future SLR exceed the 1.0 m of SLR we used within our model and therefore island responses under extreme SLR remain unknown^37^. Third, the island was exposed to continuous, rather than episodic storm conditions limiting the relaxation intervals during which islands are exposed to smaller and often constructive waves. Lastly, the rate and magnitude of sediment supply simulated during the experiments were based on storm-derived sediment supplied to Fatato Island by Hurricane Bebe in 1972^24,25,32^ (Supplementary Material S1). However, the rate and magnitude of sediment supply to *motu* is expected to vary spatially and temporally dependent on sediment generation and the frequency and magnitude of high energy events^7^.

### The effect of sediment supply on the future persistence of reef islands

Our results present the first experimental evidence that sediment supply dampens the adverse morphological effects of SLR on reef islands. The morphological outcomes observed during these experiments were similar to both conceptual and geometric modelling attempts^7,31^, which demonstrate the influence of sediment supply on the future stability of reef islands. Likewise, remote sensing observations have documented the growth response of reef islands following the input of storm-derived sediment, which in cases has grown islands beyond their pre-storm extent^17^. Our results show that by enhancing the magnitude of vertical accretion of the oceanward crest (by 15% and 42% depending on wave height), sediment supply increases the potential for islands to keep pace with SLR and offset future flood events. As well as increasing crest height, sediment supply also dampens the rate of lateral island migration and reduction in subaerial island volume and width, consequently offsetting reductions of island area. However, the extent of morphological benefits to islands from additional sediment will vary between islands depending on site-specific changes in the frequency and magnitude of sediment supply (Fig. 2).

The frequency and magnitude of storms as well as the location of storm tracks are expected to alter in response to climate change, which may lead to spatio-temporal variability in the future storm-derived sediment supply to reef islands^38,39^. Although the frequency of tropical cyclones is expected to decrease, the average global intensity of tropical cyclones is expected to increase by 2100 with substantial increases in the frequency of the most intense cyclones predicted^40,41^. Projected increases in cyclone intensity may result in a larger amount of sediment added to the sedimentary system during each event, while a reduction in storm frequency is expected to increase coral recovery time and allow the growth of new coral colonies between events^42^. These changes may reduce the susceptibility of some reef islands to SLR as increased sediment supply allows further island building and offsets the erosive effects of SLR (Fig. 1), while coral growth in between events maintains the source of sediment to the island. However, alternative changes to cyclone characteristics such as increased cyclone frequency may reduce coral recovery and growth, leading to a shutdown in sediment supply. Future island adjustments with a closed sedimentary system are expected to exacerbate erosive morphological outcomes reducing the ability of islands to offset island inundation and reduction in island area.

It is important to note that our study has examined instantaneous sediment input based on estimates of sediment generation during a hurricane in Tuvalu. The volume and composition of sediment generated by such storms will likely vary based on the ecological characteristics of the surrounding reef systems. Likewise, the future health and functionality of coral reefs remains uncertain as the effects of climate change, such as coral bleaching, on the island sedimentary system may take many years to manifest^7,43,44^. Future reductions in healthy, productive coral reefs due to increasing sea surface temperatures and ocean acidity are expected to reduce coral density and growth rates which may lead to reduced sediment generation^43^. Such changes in the health and productivity of the biological system are expected to have a substantial effect on the physical response of reef islands to SLR and changing wave conditions.

### Future physical persistence of reef islands

Our results have important implications for understanding the physical persistence of reef islands in response to increasing sea level. Results of this study suggest that islands subject to a storm-derived sediment supply are less susceptible to destabilization through wave erosion and wave-driven flooding than islands without a supply of sediment. We note, our study has examined the geomorphic response of a reef island to sea level rise and shows a potential mechanism for the geological persistence of islands. We caution that our results do not consider the habitability of reef islands, which is a function of freshwater availability, food security and myriad of other factors beyond the morphodynamic behaviour of the island. We stress the processes of wave overwash and resultant deposition of sediment on to the island surface may pose many challenges to the on-going habitability of reef islands.

**Figure 4:**
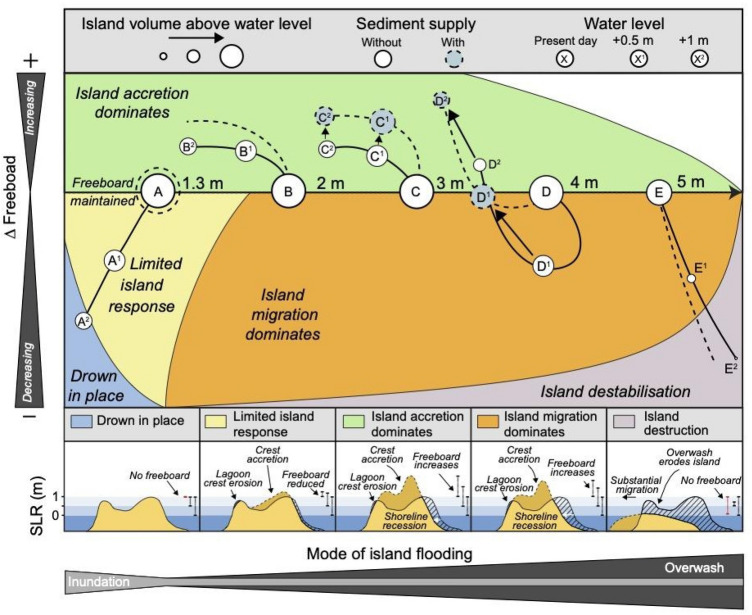
A conceptual model of the geomorphic responses of reef islands to increasing sea level and changing wave conditions with and without a sediment supply. (**A**) Reduced island response and eventual drowning (1.3 m waves), (**B**–**C**) island building (2–3 m waves), (**D**) island migration (4 m waves), and E) island destruction (5 m waves), observed during physical modelling experiments. The grey and white circles represent results presented during this study and presented in Tuck et al.^21^. Tuck et al.^21^ did not simulate sediment supply; therefore, the dashed lines without grey circles depict the conceptually modelled response of island change with a sediment supply. Black arrows show the change in positions of C and D with and without a sediment supply. SS—sediment supply.

A conceptual model of the geomorphic responses of reef islands to changing sea level and wave conditions under different amounts of sediment supply is proposed, which includes: island building, island migration as well as island drowning and destruction as the potential trajectories of island change (Fig. 4). The model identifies sediment supply as an important control on the future physical persistence of islands as sediment added to the island is expected to shift future island trajectories with SLR further towards island building and persistence on the reef platform (Fig. 4). Results demonstrate that island susceptibility to inundation is not only dependent on SLR and waves but is a product of the feedbacks between sea level, offshore wave conditions, sediment supply and the morphology of the island (Fig. 4). Islands exposed to low rates of sea-level rise, energetic wave conditions which are conducive to island building and an ample supply of sediment are expected to be at least risk of inundation from further SLR. However, the susceptibility of islands to flooding is expected to vary spatially depending on site-specific variations in the rate and magnitude of SLR, sediment supply and offshore wave conditions^5,35^.

This research improves understanding of the future physical stability of reef islands, a crucial aspect of island flood risk assessments. However, reef islands are not only at risk of being rendered uninhabitable by erosion and inundation, and factors such as access to freshwater reserves and food resources must also be considered. Our results corroborate earlier physical and numerical modelling experiments^20–22^ as well as planform island analysis^19^ that suggest the loss of land through island inundation and erosion is unlikely to render islands uninhabitable in the next few decades. Results suggest that sediment supply, when readily available, is expected to further offset the loss of land resources, increasing the future persistence of reef islands (Fig. 4).

### Implications for flood risk models

Physical models exploring the geomorphic response of reef islands to changing wave and water level conditions provide new insights for evaluating the susceptibility of islands to wave-driven flooding. Existing flood risk models that simulate future sea levels on present-day island topography are expected to overestimate flooding extent as they do not incorporate important morphological feedbacks that are expected to influence island susceptibility to SLR and flood events. Therefore, existing flood models are only appropriate to the relatively small number of islands with highly modified coastlines that are unable to geomorphically adjust to changing environmental conditions as engineering structures have locked the shoreline in place. The modelling results presented in this paper are generally applicable for *motu*, which without shoreline armouring are able to geomorphically respond to changing sea level and wave conditions by increasing elevation of the crest and island surface. Our findings show that the effect of sediment supply on the geomorphic behaviour of reef islands has substantial implications on the future physical persistence of reef islands. First, when a sediment supply was simulated in the model island freeboard was substantially increased under all SLR scenarios, which is expected to offset future increases in island flooding suggested in a number of flood risk assessments^2,13,14,16^. Second, experiments demonstrated that sediment supply also offsets the erosive effects of SLR. Of significance, modelling results demonstrate that a sediment supply dampens reductions in subaerial island volume and may explain why widespread erosion of islands has not been observed to date^45^. Third, local controls on sediment supply, SLR and storm frequency are expected to have a strong influence on variations in island susceptibility to inundation. These insights emphasize the need to incorporate both physical and ecological processes into reassessments of future wave-driven flood risk assessments in order to better resolve flood impacts and future island vulnerability.

## Methods

### Experimental setup

Experimental tests to examine the influence of sediment supply on reef island morphodynamics during rising sea levels were undertaken in a wave flume at the COAST (Coastal Ocean and Sediment Transport) lab, Plymouth University, UK. The wave flume (length: 20 m, width: 0.6 m, depth: 1 m) was equipped with one absorbing piston wave paddle and an array of 16 capacitance wave probes. A reef platform and island model were constructed within the flume at a scale of 1:50, corresponding to a Froude scaling of 1:1, representing the balance between inertial and gravitational forces and hydrodynamic similitude. Fatato Island, situated on the windward side of Funafuti atoll, Tuvalu, was chosen as the prototype island. Characteristic of *motu*, Fatato is an elongate, gravel island with an elevated oceanward berm, depressed central basin and lower elevated lagoonward berm. The construction of the model, sedimentary characteristics and wave conditions used in the laboratory experiments were based on detailed field observations at Fatato Island^20^.

**Figure 5:**
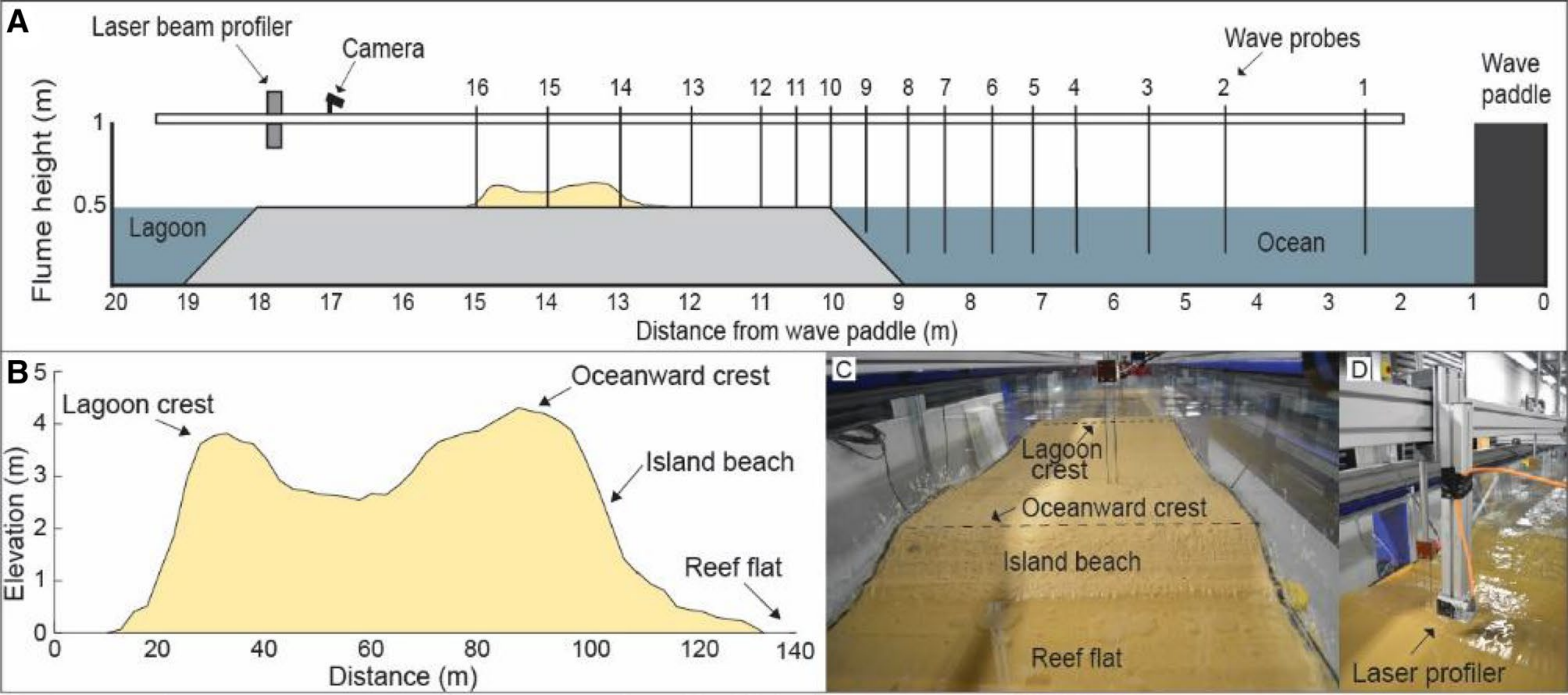
(**A**) Experimental set-up in wave flume, (**B**) surveyed cross-section of Fatato, (**C**) oblique photo of flume looking towards island beach and (**D**) photo of laser beam profiler.

The model reef platform (8 m long and 0.6 m wide) was located 0.47 m above the flume floor with a 1:2.3 forereef and backreef slope (Fig. 5). The plywood surface used as the reef platform was smooth, with minimal surface texture. In order to more closely replicate a reef flat we glued fine sand to the plywood surface to provide a degree of surface roughness. However, we note the sand does not replicate the wide range of morphological features, such as boulders, conglomerate and algal turf on the reef flat at Fatato but does offer a more realistic surface than unaltered plywood. The reef platform was positioned in the flume with the oceanward reef crest located 9 m from the face of the wave paddle (Fig. 5). The island was located 2.4 m from the reef crest and constructed out of fine sand (median = 1.5 φ; 0.35 mm). When geometrically scaled, this sand is equivalent to a grain size of 17.5 mm at prototype scale, comparable to medium gravel sediment found on Fatato Island^46^. We note that reef-derived carbonate sediments have a wide range of characteristics (i.e. size, shape and density) which control entrainment and transport^47^ and would differ based on the local sources of sediment.

For this model, the most important scale relationships were identified, and the critical parameters were kept in similitude between model and prototype^48,49^. Using the 0.35 mm sediment, it was attempted to achieve similitude for the dimensionless fall velocity, as well as the Shields and Rouse numbers, describing the threshold and mode of sediment transport, respectively. Perfect similitude between model and prototype is impossible; however, the absence of dynamic similarity between model and prototype does not detract from their usefulness with many physical models accurately replicating natural systems despite not exhibiting dynamic similarity^49^.

Wave conditions used during all the experiments were irregular and generated using a Joint North Sea Wave Project (JONSWAP) wave steering signal specified by significant wave height (H_s_), corresponding wave period (T_p_) and direction fields extracted from a 30 year WaveWatch III hindcast of a point 35 km offshore of Funafuti atoll (8°42′7.20″S 179°23′49.20″E). The hindcast^50^ provides a global wind wave climatology generated by the WaveWatch III numerical model with atmospheric forcing provided by Climate Forecast System Reanalysis (CFSR)^51^. Only onshore directed waves, approaching between 60˚ and 120˚ are taken into consideration when extracting wave parameters for Fatato Island. Wave conditions in the flume were based on the ~ 10 yr return period (3 m) and largest wave conditions (4 m) based on the Wave Watch III hindcast^50,52^. However, we note that Wave Watch III had been shown to underestimate maximum wave heights during storm conditions and it is possible that some extreme events responsible for reshaping the island may not have been captured by the model over the hindcast period. Once offshore waves had reached the reef platform in the flume the process of wave transformation across the reef were shown to closely match field observations^53,54^.

### Model validity

Tuck et al.^20^ undertook tests to assess the repeatability of the construction of the island (i.e. morphology at T_0_) and the repeatability of the geomorphic outcomes observed after exposure to waves and sea level rise in the flume. Tests were conducted under high wave energy conditions (H_s_ = 3 m) with 0.5 m of sea level rise and without sediment supply. Results showed the initial island morphology differed between tests by an average of 5.5 cm + /− 11.8 cm (2 std. dev.) at prototype scale (1.10 mm + /− 2.36 mm unscaled), showing the ability to accurately reconstruct the island morphology using the templates. Similarly, the final morphology of the island, after exposure to waves and SLR for 30 min, was 6.9 cm + /− 13.95 cm (2 std. dev.) (1.38 mm + /- 2.79 mm unscaled).

### Experimental programme

The morphological response of the island to changing incidents waves, SLR and sediment supply was examined in four experimental series each comprising three 180 min runs (Supplementary Table 1). The four experiment series involved running the model with 3 m and 4 m waves both with and without sediment supply. Within each experiment series the flume was run three times for 180 min at present day water level with subsequent increases in water level to replicate 0.5 m and 1.0 m of SLR above present day. At the beginning of each experiment series, the scaled island was reconstructed on the reef platform using a template to ensure the island was accurately recreated.

During experiments which included the addition of 800 cm^3^ (2 kg) of sediment, equivalent to 2% of the island volume, was added to the island shoreline every 60 min (Supplementary Table 1; Supplementary Material S1). This amount of material represents a significant input to the island sedimentary system; however, it is well below the amount of sediment deposited on the reef platform at Fatato Island following Hurricane Bebe in 1972 which increased the area of Fatato by 10%^55^. During the experimental runs water level in the flume was set at present-day spring high tide level and was increased at 10 mm increments every 180 min to achieve SLR at prototype scales of 0.5 m and 1.0 m above spring high tide (Supplementary Table 1). The island’s morphology was surveyed before and after each experiment and at 60-min intervals during each test. Each survey measured along the central island using a laser beam profiler at 0.05 m horizontal increments. Volume estimates based on a 30 m cross-section of the island as well as island width, crest height, crest migration and centre of mass calculated for each profile were used to assess characteristics of morphological change.

## Supplementary Information

Supplementary Information 1.

Supplementary Information 2.

## Data Availability

Data reported in the paper are available in the supplementary online information.

## References

[1] Albert S, Leon JX, Grinham AR, Church JA, Gibbes BR, Woodroffe CD (2016). Interactions between sea-level rise and wave exposure on reef island dynamics in the Solomon Islands. Environ. Res. Lett..

[2] Storlazzi CD, Elias EPL, Berkowitz P (2015). Many atolls may be uninhabitable within decades due to climate change. Nat. Sci. Rep..

[3] Kopp RE (2014). Probabilistic 21st and 22nd century sea-level projections at a global network of tide-gauge sites. Earth’s Future.

[4] Deconto RM, Pollard D (2016). Contribution of Antarctica to past and future sea-level rise. Nature.

[5] Nurse LA, Barros VR, Field CB, Dokken DJ, Mastrandrea MD, Mach KJ, Bili TE, Chatterjee M, Ebi KL, Estrada YO, Genova RC, Girma B, Kissel ES, Levy AN, MacCracken S, Mastrandrea PR, White LL (2014). Small islands. Climate Change 2014: Impacts, Adaptation, and Vulnerability. Part B: Regional Aspects. Contribution of Working Group II to the Fifth Assessment Report of the Intergovernmental Panel on Climate Change.

[6] Shope JB, Storlazzi CD, Erikson LH, Hegermiller CA (2016). Changes to extreme wave climates of islands within the Western Tropical Pacific throughout the 21st century under RCP 4.5 and RCP 8.5, with implications for island vulnerability and sustainability. Global Planet. Change.

[7] Perry CT (2011). Implications of reef ecosystem change for the stability and maintenance of coral reef islands. Glob. Change Biol..

[8] Dawson JL, Smithers SG, Hua Q (2014). The importance of large benthic foraminifera to reef island sediment budget and dynamics at Raine Island, northern Great Barrier Reef. Geomorphology.

[9] Kench P, Perry C, Spencer T, Slaymaker O, Spencer T, Embleton-Hamann C (2009). Coral reefs. Geomorphology and Global Environmental Change.

[10] Richmond, B. M. Development of atoll islets in the central Pacific Ocean. *Proceeding of the 7th International Coral Reef Symposium, Guam*, **2**, 1185–1194 (1992).

[11] Stoddart DR, Steers JA, Jones OA, Endean R (1997). The nature and origin of coral reef islands. Biology and Geology of Coral Reefs.

[12] Grady AE, Moore LJ, Storlazzi CD, Elias E, Reidenbach MA (2013). The influence of sea level rise and changes in fringing reef morphology on gradients in alongshore sediment transport. Geophys. Res. Lett..

[13] Storlazzi CD (2018). Most atolls will be uninhabitable by the mid-21st century because of sea-level rise exacerbating wave-driven flooding. Sci. Adv..

[14] Beetham E, Kench PS (2018). Predicting wave overtopping thresholds on coral reef-island shorelines with future sea-level rise. Nat. Commun..

[15] Weiss KR (2015). Before we drown we may die of thirst. Nature News.

[16] Merrifield M, Becker J, Ford M, Yao Y (2014). Observations and estimates of wave-driven water level extremes at the Marshall Islands. Geophys. Res. Lett..

[17] Ford MR, Kench PS (2016). Spatiotemporal variability of typhoon impacts and relaxation intervals on Jaluit Atoll, Marshall Islands. Geology.

[18] Duvat VKE, Pillet V (2017). Shoreline changes in reef islands of the Central Pacific: Takapoto Atoll, Northern Tuamotu, French Polynesia. Geomorphology.

[19] Kench PS, Ford MR, Owen SD (2018). Patterns of island change and persistence offer alternate adaptation pathways for atoll nations. Nat. Commun..

[20] Tuck ME, Kench PS, Ford MR, Masselink G (2019). Physical modelling of the response of reef islands to sea-level rise. Geology.

[21] Tuck ME, Ford MR, Masselink G, Kench PS (2019). Physical modelling of reef island topographic response to rising sea levels. Geomorphology.

[22] Masselink G, Beetham E, Kench P (2020). Coral reef islands can accrete vertically in response to sea level rise. Sci. Adv..

[23] Beetham E, Kench PS, Popinet S (2017). Future reef growth can mitigate physical impacts of sea level rise on atoll islands. Earth’s Future.

[24] Bayliss-Smith TP (1988). The role of hurricanes in the development of reef islands, Ontong Java Atoll, Solomon Islands. Geogr. J..

[25] Maragos JE, Baines GBK, Beveridge PJ (1973). Tropical cyclone creates a new land formation on Funafuti atoll. Science.

[26] Kayanne H (2016). Eco-geomorphic processes that maintain a small coral reef island: Ballast Island in the Ryukyu Islands, Japan. Geomorphology.

[27] Smithers SG, Hoeke RK (2014). Geomorphological impact of high-latitude storm waves on low-latitude reef islands: Observations of the December 2008 event on Nukutoa, Takuu Papua New Guinea. Geomorphology.

[28] Ford MR, Kench PS (2014). Formation and adjustment of typhoon-impacted reef islands interpreted from remote imagery: Nadikdik Atoll, Marshall Islands. Geomorphology.

[29] Kench PS (2006). Geological effects of tsunami on mid-ocean atoll islands: the Maldives before and after the Sumatran tsunami. Geology.

[30] Kench PS, Nichol SL, Smithers SG, Mclean RF, Brander RW (2008). Tsunami as agents of geomorphic change in mid-ocean reef islands. Geomorphology.

[31] Kench, P. S. & Cowell, P. J. Variations in sediment production and implications for atoll island stability under rising sea level. In *Proceedings of 9th International Coral Reef Symposium,* Bali, 2, 1181–1186 (2002).

[32] Baines GB, McLean RF (1976). Sequential studies of hurricane deposit evolution at Funafuti Atoll. Mar. Geol..

[33] Harris DL, Rovere A, Casella E, Power H, Canavesio R, Collin A, Pomeroy A, Webster JM, Parravicini V (2018). Coral reef structural complexity provides important coastal protection from waves under rising sea levels. Sci. Adv..

[34] Perry CT (2018). Loss of coral reef growth capacity to track future increases in sea level. Nature.

[35] Church JA, Stocker T, Qin D, Plattner G, Tignor M, Allen S, Boschung J, Nauels A, Xia Y, Bex V, Midgley P (2013). Sea level change. Climate Change 2013: The Physical Science Basis. Contribution of Working Group I to the Fifth Assessment Report of the Intergovernmental Panel on Climate Change.

[36] Siegert M, Alley RB, Rignot E, Englander J, Corell R (2020). Twenty-first century sea-level rise could exceed IPCC projections for strong-warming futures. One Earth.

[37] Garner AJ, Weiss JL, Parris A, Kopp RE, Horton RM, Overpeck JT, Horton BP (2018). Evolution of 21st century sea level rise projections. Earth's Future.

[38] Shaw TA (2016). Storm track processes and the opposing influences of climate change. Nat. Geosci..

[39] Magee AD, Lorrey AM, Kiem AS, Colyvas K (2020). A new island-scale tropical cyclone outlook for southwest Pacific nations and territories. Scientific reports.

[40] Knutson TR (2010). Tropical cyclones and climate change. Nat. Geosci..

[41] Bacmeister JT (2018). Projected changes in tropical cyclone activity under future warming scenarios using a high-resolution climate model. Clim. Change.

[42] Puotinen M, Maynard JA, Beeden R, Radford B, Williams GJ (2016). A robust operational model for predicting where tropical cyclone waves damage coral reefs. Sci. Rep..

[43] Hoegh-Guldberg O (2007). Coral reefs under rapid climate change and ocean acidification. Science.

[44] Hughes TP (2017). Global warming and recurrent mass bleaching of corals. Nature.

[45] Duvat VKE (2019). A global assessment of atoll island planform changes over the past decades. WIREs Clim. Change.

[46] Ryan, E. J. The Nearshore Process Regime Around an Atoll Motu and Implications for Beach Morphodynamics: A case study of Fatato, Funafuti Atoll, Tuvalu [Masters thesis]. Auckland, University of Auckland, 131p. (2012).

[47] Riazi A, Vila-Concejo A, Salles T, Türker U (2020). Improved drag coefficient and settling velocity for carbonate sands. Sci. Rep..

[48] Dean RG, Dalrymple RA (2004). Coastal Processes with Engineering Applications.

[49] Paola C, Straub K, Mohrig D, Reinhardt L (2009). The, “unreasonable effectiveness” of stratigraphic and geomorphic experiments. Earth Sci. Rev..

[50] Hemer MA, Fan Y, Mori N, Semedo A, Wang XL (2013). Projected changes in wave climate from a multi-model ensemble. Nat. Clim. Chang..

[51] Tolman, H. L. Usermanual and system documentation of WAVEWATCH III version 3.14. NOAA/NWS/NCEP/MMAB Technical Note 276, 19 (2009).

[52] Bosserelle C, Reddy S, Lal D (2015). WACOP Wave Climate Reports.

[53] Tuck ME, Ford MR, Masselink G, Kench PS (2018). Physical modelling of reef platform hydrodynamics. J. Coast. Res..

[54] Masselink G, Tuck M, McCall R, van Dongeren A, Ford M, Kench P (2019). Physical and numerical modelling of infragravity wave generation and transformation on coral reef platforms. J. Geophys. Res. Oceans.

[55] Woodroffe CD (2008). Reef-island topography and the vulnerability of atolls to sea-level rise. Glob. Planet. Chang..

