# Supplementary material for: Sediment supply dampens the erosive effects of sea-level rise on reef islands
Source: Sci Rep. 2021 Mar 9;11:5523. doi: 10.1038/s41598-021-85076-x (PMC7970901; doi:10.1038/s41598-021-85076-x)
Supplement: Supplementary file 1 — Supplementary Information 1. [file 41598_2021_85076_MOESM1_ESM.docx]

Sediment supply dampens the erosive effects of sea-level rise on reef islands.

Megan E. Tuck^1^, Murray R. Ford^1^, Paul S. Kench^2^ & Gerd Masselink^3^

^1^School of Environment, University of Auckland, Private Bag 92019, Auckland, New Zealand. [mtuc652@aucklanduni.ac.nz](about:blank), [m.ford@auckland.ac.nz](about:blank)

^2^Department of Earth Sciences, Simon Fraser University, BC, Canada
[pkench@sfu.ca](about:blank)

^3^School of Biological and Marine Sciences, University of Plymouth, PL4 8AA, Plymouth, UK
[g.masselink@plymouth.ac.uk](about:blank)

Corresponding author: Megan Tuck - [mtuc652@aucklanduni.ac.nz](about:blank)

Supplementary Material S1: Determining sediment supply to model island

The physical modelling experiments presented in this paper explore the effect of a storm derived sediment supply on the geomorphic behaviour of islands in response to SLR and energetic wave conditions. The volume of sediment added to the island was determined by the magnitude of sediment added to Fatato Island, Funafuti Atoll, Tuvalu, during Cyclone Bebe in 1972. During the storm, extreme wave conditions dislodged coral material from the forereef and deposited it as a large rubble rampart along the south-eastern reef platform of Funafuti Atoll. The rampart was subsequently reworked and transported across the reef platform by lower magnitude wave conditions and eventually joined Fatato, increasing the area of the island by approximately 10% [1,2,3] (Fig. 1).


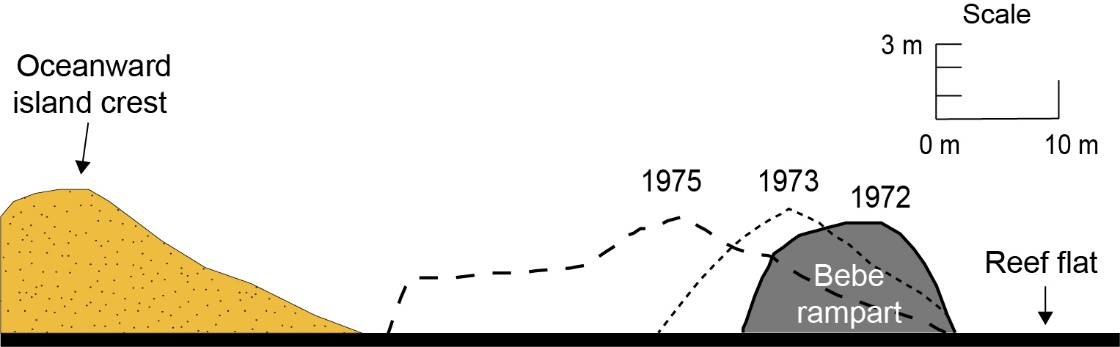


Fig.1: Bebe rampart migration towards Fatato Island, Funafuti Atoll, Tuvalu. Adapted from Baines and McLean, [2].

A number of studies have recorded substantial increases in island area and volume as a result of sediment deposited on the reef platform by high energy events [4,5,6,7,8]. Duvat and Pillet [7] calculated that Tropical Cyclone Orama contributed up to 62% of sediment for island accretion on Takapoto Atoll over the last 30 years [7]. Ford and Kench [5] found similar constructive impacts of high energy events after exploring the longer-term geomorphic island response at Jaluit Atoll, Republic of the Marshall Islands to Typhoon Ophelia in 1958. Results from remote sensing analysis indicates that the rubble bars deposited by Typhoon Ophelia in 1958 have driven island accretion on Jaluit Atoll with total landmass now exceeding pre-Ophelia area.

In order to explore the effect of a storm derived sediment supply on the geomorphic behaviour of reef islands 800 cm^3^ (2 kg) of sediment, equivalent to 2% of the island volume, was added was added to the island beach every 60 minutes during experiment series 3 and 4 (Supplementary Table 1). The amount of sediment added to the island during each experiment is well within reasonable parameters, representing a substantial input to the sedimentary system but well below the amount of sediment deposited on Fatato Island during Cyclone Bebe in 1972. It is important to note that the volume and frequency of sediment supply added to the island during the experiments was not chosen to recreate a particular environmental scenario, but to explore the effect of a sediment supply on the modes and styles of island response to increasing sea level and energetic wave conditions.

**References**

1. Maragos, J. E., Baines, G. B. K. & Beveridge, P. J. Tropical cyclone creates a new land formation on Funafuti atoll. *Science*, **181**, 1161–1164 (1973).
2. Baines, G. B., & McLean, R. F. Sequential studies of hurricane deposit evolution at Funafuti Atoll. *Marine Geology*, **21** (1), M1-M8 (1976).
3. Woodroffe, C.D. Reef-island topography and the vulnerability of atolls to sea-level rise. *Glob. Planet. Chang*. **62**, 77-96, <https://doi.org/10.1016/j.gloplacha.2007.11.001> (2008).
4. Blumenstock, D. I., Fosberg, F. R. & Johnson, C. G. The re-survey of typhoon effects on Jaluit Atoll in the Marshall Islands. *Nature*, **189**, 618-620 (1961).
5. Ford, M.R. & Kench, P.S. Spatiotemporal variability of typhoon impacts and relaxation intervals on Jaluit Atoll, Marshall Islands, *Geology* **44** (2), 159-162, <http://dx.doi.org/10.1130/G37402.1> (2016).
6. Kayanne, H. *et al.* Eco-geomorphic processes that maintain a small coral reef island: Ballast Island in the Ryukyu Islands, Japan. *Geomorphology* **271**, 84–93, <https://doi.org/10.1016/j.geomorph.2016.07.021> (2016).
7. Duvat, V.K.E. & Pillet, V. Shoreline changes in reef islands of the Central Pacific: Takapoto Atoll, Northern Tuamotu, French Polynesia: *Geomorphology*, **282**, 96–118, (2017).
8. Kench, P. S., McLean, R. F., Owen, S. D., Tuck, M. & Ford, M. R. Storm-deposited coral blocks: A mechanism of island genesis, Tutaga Island, Funafuti atoll, Tuvalu. *Geology*, **46**, 915–918 (2018).
