# Supplementary material for: Sediment supply dampens the erosive effects of sea-level rise on reef islands
Source: Sci Rep. 2021 Mar 9;11:5523. doi: 10.1038/s41598-021-85076-x (PMC7970901; doi:10.1038/s41598-021-85076-x)
Supplement: Supplementary file 2 — Supplementary Information 2. [file 41598_2021_85076_MOESM2_ESM.docx]

Sediment supply dampens the erosive effects of sea-level rise on reef islands.

Megan E. Tuck^1^, Murray R. Ford^1^, Paul S. Kench^2^ & Gerd Masselink^3^

^1^School of Environment, University of Auckland, Private Bag 92019, Auckland, New Zealand. [mtuc652@aucklanduni.ac.nz](about:blank), [m.ford@auckland.ac.nz](about:blank)

^2^Department of Earth Sciences, Simon Fraser University, BC, Canada
[pkench@sfu.ca](about:blank)

^3^School of Biological and Marine Sciences, University of Plymouth, PL4 8AA, Plymouth, UK
[g.masselink@plymouth.ac.uk](about:blank)

Corresponding author: Megan Tuck - [mtuc652@aucklanduni.ac.nz](about:blank)

Supplementary Table 1

**Supplementary Table 1**: Hydrodynamic parameters used within the Wave flume experiments. Exp. = experiment run, H_s_ = significant wave height, h_reef_ = water level on reef, T_p_ = wave period, T_test_ = test time. Prototype vales are given in parentheses.

| Exp. | *H_s_* (m) | *h_reef_* (m) | *T_p_* (seconds) | *T_test_* (min) | Sediment supply |
| --- | --- | --- | --- | --- | --- |
| Exp 1.1 | 0.06 (3) | 0.040 (2.0) | 1.4 (9.9) | 180 | No |
| Exp 1.2 | 0.06 (3) | 0.045 (2.5) | 1.4 (9.9) | 180 | No |
| Exp 1.3 | 0.06 (3) | 0.050 (3.0) | 1.4 (9.9) | 180 | No |
| Exp 2.1 | 0.06 (3) | 0.040 (2.0) | 1.4 (9.9) | 180 | Yes |
| Exp 2.2 | 0.06 (3) | 0.045 (2.5) | 1.4 (9.9) | 180 | Yes |
| Exp 2.3 | 0.06 (3) | 0.050 (3.0) | 1.4 (9.9) | 180 | Yes |
| Exp 3.1 | 0.08 (4) | 0.040 (2.0) | 1.4 (9.9) | 180 | No |
| Exp 3.2 | 0.08 (4) | 0.045 (2.5) | 1.4 (9.9) | 180 | No |
| Exp 3.3 | 0.08 (4) | 0.050 (3.0) | 1.4 (9.9) | 180 | No |
| Exp 4.1 | 0.08 (4) | 0.040 (2.0) | 1.4 (9.9) | 180 | Yes |
| Exp 4.2 | 0.08 (4) | 0.045 (2.5) | 1.4 (9.9) | 180 | Yes |
| Exp 4.3 | 0.08 (4) | 0.050 (3.0) | 1.4 (9.9) | 180 | Yes |
